# Argonaute Proteins: From Structure to Function in Development and Pathological Cell Fate Determination

**DOI:** 10.3389/fcell.2019.00360

**Published:** 2020-01-22

**Authors:** Madlen Müller, Francesco Fazi, Constance Ciaudo

**Affiliations:** ^1^Swiss Federal Institute of Technology Zurich, Department of Biology, IMHS, Zurich, Switzerland; ^2^Life Science Zurich Graduate School, Molecular Life Sciences Program, University of Zurich, Zurich, Switzerland; ^3^Department of Anatomical, Histological, Forensic & Orthopedic Sciences, Section of Histology & Medical Embryology, Sapienza University of Rome, Laboratory Affiliated to Instituto Pasteur Italia-Fondazione Cenci Bolognetti, Rome, Italy

**Keywords:** argonaute proteins, expression, structure, posttranslational modifications, development, cancer

## Abstract

The highly conserved Argonaute protein family members play a central role in the regulation of gene expression networks, orchestrating the establishment and the maintenance of cell identity throughout the entire life cycle, as well as in several human disorders, including cancers. Four functional Argonaute proteins (AGO1–4), with high structure similarity, have been described in humans and mice. Interestingly, only AGO2 is robustly expressed during human and mouse early development, in contrast to the other AGOs. Consequently, AGO2 is indispensable for early development *in vivo* and *in vitro*. Here, we review the roles of Argonaute proteins during early development by focusing on the interplay between specific domains of the protein and their function. Moreover, we report recent works highlighting the importance of AGO posttranslational modifications in cancer.

## Introduction

Historically, the Argonaute (AGO) protein family has been discovered in a plant mutagenesis screen, performed to identify new genes involved in *Arabidopsis thaliana* development (Bohmert et al., 1998). This first report already highlighted the conservation of the Argonaute gene family in multicellular organisms suggesting its important functions. It was later demonstrated that AGOs are conserved throughout all domains of life (Swarts et al., 2014). Eukaryotic AGOs are involved in many cellular processes and act as mediators of gene silencing (Bartel, 2018). In mammals, AGOs have been mainly described for their cytoplasmic role in small RNA (smRNA) biogenesis, as key components of the RNA-induced silencing complex (RISC) (Bodak et al., 2017a).

Two types of ∼22 nt smRNAs can be loaded into AGOs to induce translational inhibition or exonucleolytic messenger RNA (mRNA) decay of specific transcripts: small interfering RNAs (siRNAs) and microRNAs (miRNAs). Both species are processed in the cytoplasm by DICER, leading to the release of double-stranded RNA (dsRNA) duplexes, which will be loaded into the RISC complex to achieve its RNA interference (RNAi) functions [for reviews (Bodak et al., 2017a; Treiber et al., 2019)].

Furthermore, the regulatory role of smRNAs expands beyond the posttranscriptional regulation mediated by miRNAs. In fact, smRNAs with AGOs as their effector proteins have been described to be involved in transcriptional gene silencing or activation (Malecová and Morris, 2010), alternative splicing (Alló et al., 2009; Harel-Bellan et al., 2013), antiviral defense (Maillard et al., 2013), genome integrity control (Svoboda et al., 2004; Kanellopoulou et al., 2005; Bodak et al., 2017b), DNA repair (Hawley et al., 2017), and epigenetic modification of the chromatin (Li, 2014). Although the expression of new smRNA species such as small nucleolar RNA (sno-RNA)- and transfer RNA (tRNA)-derived fragments has been recently described to be altered in the context of cancer, their functions remain largely unexplored (Martens-Uzunova et al., 2013; Schorn et al., 2017; Kuscu et al., 2018). However, recent evidence shows that the functions and biogenesis of these new smRNA species are tightly connected to the RNAi pathway, functioning both in the cytoplasm and the nucleus (Huang and Li, 2014; Sarshad et al., 2018).

In this review, we highlight novel findings on the structures of AGO proteins since the description of the human AGO2 (Schirle and Macrae, 2012) and link these with their roles in mammalian early development and carcinogenesis.

## Structure and Domains of the Argonaute Proteins

Structures of prokaryotic and mammalian Argonaute proteins have been extensively studied in the past decades and have given revealing insights into the mechanism of translational inhibition by miRNAs. In this part, we only focus on the structural data of the human AGO proteins, which are highly conserved and share ∼85% of sequence identity (https://myhits.isb-sib.ch/cgi-bin/profile_search?data=5485215623128241).

Four Argonaute proteins (AGO1–4) are expressed in humans. AGO2 is described best and has long been thought to be the only Argonaute protein member having mRNA slicing activity, due to its unique structural characteristics (Liu et al., 2004; Meister et al., 2004). Nevertheless, AGO3 has recently been shown to slice target RNAs, however, only when loaded with certain miRNAs (Park et al., 2017). In these cases compared to AGO2, the slicing activity depended strongly on the pairing of the postseed region of the guide RNA as well as on the 5′ and 3′ flanking regions of the target RNA (Park et al., 2017).

**FIGURE 1:**
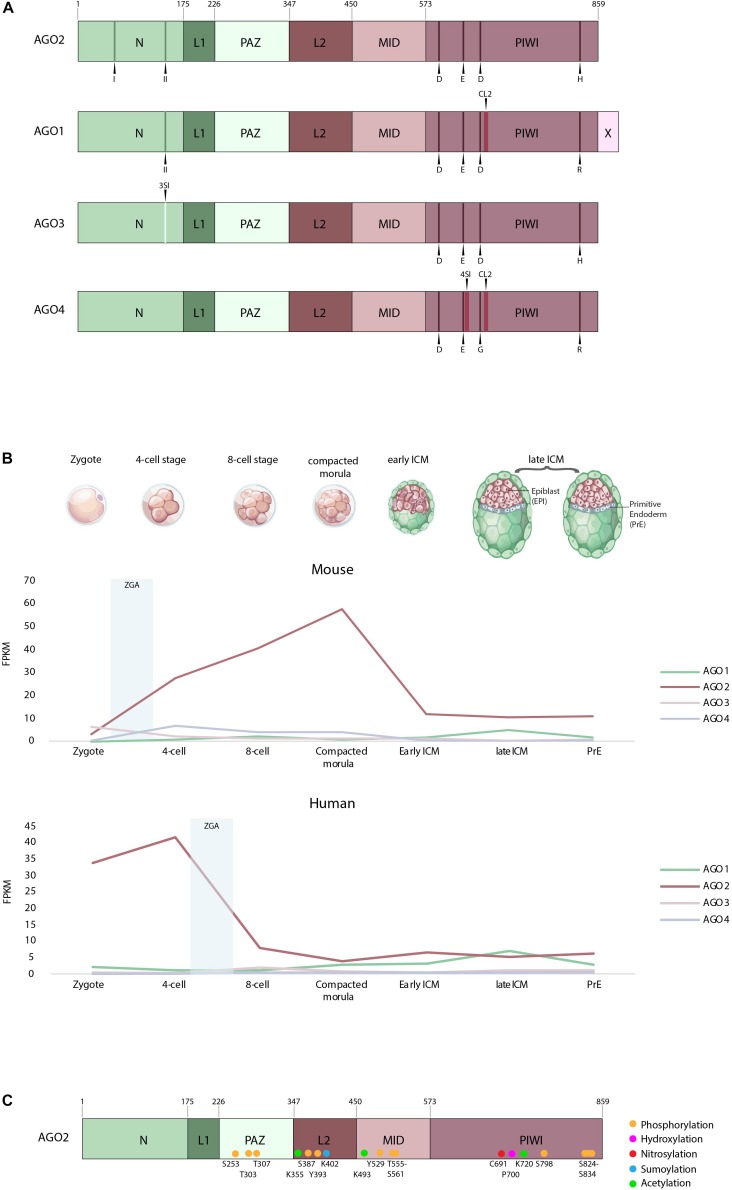
**(A)** Domain organization of AGO1–4 (adapted from Elkayam et al., 2012). Indicated are the two N-terminal motifs labeled I and II and the residues of the catalytic tetrad in the PIWI domain D, E, D, H, R, and G. Also highlighted in AGO1 and AGO4 is cluster 2 (CL2), the AGO3-specific insertion (3SI) and the AGO4-specific insertion (4SI). N, N-terminus; L1, linker domain 1; PAZ, PIWI/Argonaute/Zwille domain; L2, linker domain 2; MID, MID domain; PIWI, P-element-induced whimpy testes domain; X, AGO1x additional 33 aa; D, aspartate; E, glutamate; H, histidine; R, arginine; G, glycine. **(B)** Expression of human and mouse AGO1–4 in the zygote, four-cell, eight-cell, compacted morula, early inner cell mass (ICM) and late ICM, according to single-cell expression data from Boroviak et al. (2018). ZGA, first major wave of zygotic gene activation. **(C)** Posttranslational modifications of the human Argonaute 2 protein.

The four human Argonaute proteins are structurally very similar but nevertheless contain few non-conserved amino acids in their functional domains. The AGO2 full-length protein structure was resolved first and was largely studied (Elkayam et al., 2012; Schirle and Macrae, 2012; Figure 1A). Since then, structural data on all the others, AGO1, 3, 4 full proteins have become available (Faehnle et al., 2013; Nakanishi et al., 2013; Park et al., 2017, 2019). These studies have revealed four conserved domains: the N-terminal domain (N), the PIWI/Argonaute/Zwille (PAZ) domain, the MID domain, and the P-element-induced whimpy tested (PIWI) domain. The PAZ domain, which is required for anchoring the 3′ end of guide RNAs, and the MID domain, which binds the 5′ phosphate of guide RNAs (Lingel et al., 2003; Song et al., 2003, 2004; Ma et al., 2004; Yan et al., 2004; Boland et al., 2010, 2011; Frank et al., 2010), are very similar between the four AGOs (Figure 1A).

The N-terminal domain, however, differs between AGO1–4. In AGO2, the N-terminal domain comprises two motifs (residues 44–48 and 134–166), which are required for its full catalytic activity. Upon mutation of these motifs, AGO2 fails to initiate RISC activation and mRNA cleavage. During protein folding, these residues are located in the vicinity of the PIWI domain and hence are required for correct guide-target positioning (Hauptmann et al., 2013; Schürmann et al., 2013; Figure 1A). On the other hand, AGO1 harbors only one of the N-terminal motifs, required for full catalytic activity (Faehnle et al., 2013; Hauptmann et al., 2013, 2014; Figure 1A), whereas AGO3 and AGO4 possess none, which was thought to render them catalytically inactive (Faehnle et al., 2013; Hauptmann et al., 2013, 2014; Nakanishi et al., 2013; Schürmann et al., 2013; Park et al., 2019). In addition, AGO3 has a specific insertion (3SI) in the N-terminal domain, which leads to a wider and imperfect nucleic-acid binding channel compared to AGO2 (Park et al., 2017).

The PIWI domain is similar to an RNAse H domain, harboring the catalytic triad DDH, which is critical for the slicing activity of AGO2 (Parker et al., 2004, 2005; Song et al., 2004; Ma et al., 2005; Rivas et al., 2005; Yuan et al., 2005). This work has later been challenged by Nakanishi et al. (2012), who demonstrated that not only is a catalytic triad but also a catalytic tetrad (DEDH) is essential for the AGO2 slicing activity (Figure 1A). Indeed, mutation of the glutamate in this catalytic tetrad abolishes the ability of the protein to induce RNAi (Nakanishi et al., 2012). AGO3, like AGO2, has a fully functional PIWI domain with a DEDH. The slicing activity of this domain has been proven by domain swap experiments, showing that AGO3 PIWI domain introduced in an AGO2–AGO3 PIWI chimeric protein can be catalytically active (Hauptmann et al., 2013; Schürmann et al., 2013). AGO1 comprises also several domain changes, the first one being a residue change in the catalytic tetrad of the PIWI domain (Figure 1A). Second, two proline residues at position 670 and 675 in the unique structural element, called cluster 2 (CL2) [also known as conserved segment (CS7)] can bend the protein, which sterically hinders the positioning of the guide/target complex (Nakanishi et al., 2013). In the same conserved segment, another mutated residue, L674, has been shown to decrease the slicing efficiency of AGO1 (Faehnle et al., 2013). Similar to AGO1, AGO4 lacks key catalytic residues and has an additional AGO4-specific insertion (4SI) in the PIWI domain, together with the cluster 2, already observed in AGO1. Only swapping of these domains with their AGO2 counterparts has enabled AGO4 to be catalytically active. Therefore, the native AGO4 is thought to be slicing incompetent (Nakanishi et al., 2013; Hauptmann et al., 2014; Park et al., 2019; Figure 1A). In addition, in the recently published AGO4 structure, the so-called LAKEs were observed, which are an accumulation of water molecules below the nucleic acid binding channel. This formation is conserved in all human AGOs. LAKE formation aids to establish the RISC assembly and is important for smRNA duplex loading (Park et al., 2019).

Finally, AGO1 has also been detected as a candidate for programed translational readthrough, a process generating longer isoforms by continuing translation beyond the stop codon (Eswarappa et al., 2014). Two recent studies demonstrated the presence of this translational readthrough product of AGO1 in cells, termed AGO1x (Ghosh et al., 2019; Singh et al., 2019). AGO1x is a protein isoform, which is 33 amino acids longer than AGO1 (Figure 1A). Initially, it was shown in HeLa cells that AGO1x can interact with miRNAs and their mRNA targets; however, no interaction with GW182 has been observed. GW182 proteins normally interact with the Argonautes, mediating translational repression (Eulalio et al., 2009). Since AGO1x is incapable of interacting with GW182, it cannot induce translational repression. It is therefore thought that AGO1x competes with the canonical miRNA pathway and thereby leads to reduced posttranscriptional repression of target mRNAs (Singh et al., 2019). Second, in breast cancer cells, AGO1x has been shown to prevent the accumulation of dsRNAs and thereby suppresses the interferon response in these cells, a function independent of the canonical miRNA pathway (Ghosh et al., 2019).

## Expression of the Argonaute Proteins in Early Development

Although structurally very similar, the expression of AGOs can differ greatly during early development. We focus in this part on the difference in expression of mammalian AGO1–4 during early embryonic development. The mouse and human AGOs are highly conserved with almost identical protein sequences [99% for AGO2, 3, and 4 and 100% for AGO1 between mouse and human (https://myhits.isb-sib.ch/cgi-bin/profile_search?data=5485215623128241)].

The expression of the four AGOs during mouse early development was originally monitored using reverse transcription followed by PCR approaches and revealed the expression of the four transcripts in oocytes and at early stages of development (Lykke-Andersen et al., 2008). Nowadays, newer technologies allow to determine the expression of certain transcripts on a single-cell level (Stuart and Satija, 2019). A recent study, using this single-cell sequencing technology at different stages of preimplantation development in humans and mice allowed us to look into the detailed expression of the Argonautes throughout preimplantation development (Boroviak et al., 2018). Two cell fate decision events occur during preimplantation development (Niakan and Eggan, 2013). At the blastocyst stage, two populations of cells are segregating to create two distinct lineages: the trophectoderm, an extraembryonic tissue at the origin of the placenta, and the inner cell mass (ICM), the future epiblast at the origin of the three germ layers of the embryo. This first cell fate choice takes place 3 days postfertilization (dpf) in mice and 5 dpf in humans. The second cell fate specification event allows the segregation of the ICM and another extraembryonic layer: the primitive endoderm at the origin of the yolk sac, which appears 4 and 7 dpf in mice and humans, respectively (Niakan and Eggan, 2013).

For both species, the monitored expression of *Ago*1, 3, and 4 mRNAs during preimplantation stages is low compared to *Ago*2 transcripts (Figure 1B). However, it is to note that, in both species, *Ago2* represents still <1% of all detected transcripts (Boroviak et al., 2018). In mouse, *Ago*1, 3, and 4 mRNAs are lowly expressed from the zygote to the early ICM and the primitive endoderm, compared to *Ago*2 (Figure 1B). Interestingly, at the late blastocyst stage, a decrease in *Ago*2 is observed, in parallel with an increase in *Ago*1, suggesting a possible novel role for AGO1 just before implantation (Figure 1B). The expression profiles of the AGOs seem very different in human early embryos. *AGO1* mRNAs increase continuously from the eight-cell stage to reach comparable levels of *AGO2* expression, or even slightly higher at the late ICM stage (Figure 1B). In both species, however, *AGO2* is the most expressed Argonaute mRNA during early preimplantation development, in embryonic and primitive endoderm lineages. However, maximal expression occurs at different stages in human and mouse. Human *AGO2* (*hAGO2*) peaks at the four-cell stage, whereas mouse *Ago*2 (*mAgo2*) peaks at the morula stage (Figure 1B). As AGOs are actually required in early stages to degrade maternally deposited transcripts (Lykke-Andersen et al., 2008), the question arises whether the expression of *AGO2* coincides with zygotic gene activation (ZGA). In humans, the first major wave of ZGA occurs at the 4- to 8-cell stages followed by a second one at the 8- to 16-cell stages [for review, see Jukam et al. (2017)]. This, however, does not anymore correlate with the expression of h*AGO2* transcripts, which decreases after the four-cell stage. Moreover, it raises the question whether most of the *hAGO2* in early development is actually maternally deposited. On the contrary, the first major wave of ZGA in mice is already detectable at the two-cell stage, followed by a second wave at the four- to eight-cell stages [for review, see Jukam et al. (2017)]. This correlates with the increasing expression of m*Ago2*, which reaches its peak at the morula stage. Interestingly, recent studies in mice preimplantation development have demonstrated that mRNA levels do not always correlate with the protein levels (Gao et al., 2017). Moreover, protein expression often lags behind the mRNA expression in the process of ZGA. Therefore, we do not know whether the mRNA expression levels of the Argonautes discussed above reflect the protein levels within each cell (Gao et al., 2017).

### Posttranslational Modifications of Argonaute Proteins and Their Functions

Posttranslational modifications (PTMs) of proteins mediate a huge range of signaling events within a cell and are therefore critical for distinct processes such as developmental timing (Seet et al., 2006). In fact, in *Caenorhabditis elegans*, phosphorylation of the ALG-1 protein, an ortholog of the human Argonaute proteins, is required for miRNA-mediated gene silencing and the proper animal development (Quévillon Huberdeau et al., 2017). Animals expressing a phosphomutant ALG-1 display developmental defects and die at the adult stage, exemplifying the importance of posttranslational modification of Argonaute proteins during development (Quévillon Huberdeau et al., 2017).

AGO2 has been shown to be highly posttranslationally modified, which affects its protein stability and miRNA activity (Figure 1C) (Meister, 2013). Most of the posttranslational modifications have been observed in human cancer cells, yet their amino acids are conserved between mice and humans. The regulation of PTM of AGO proteins was recently related to the activity of well-characterized oncogenes, underlining the relevance of AGO-dependent pathways deregulation in cancer development. However, their importance in early development has not been assessed in mammals. In Table 1, we highlight the reported PTM sites of AGO2 and their molecular consequences.

**TABLE 1:** Posttranslational modifications of the human Argonaute 2 protein (Jee and Lai, 2014; Gebert and MacRae, 2019).

**Posttranslational modifications**	**Conserved between hAGO1-4**	**Molecular functions**	**Cellular system identified**	**References**
P700 Hydroxylation	Yes	• Increases AGO2 stability • Increases RISC function	HEK-293T, HeLa S3, U2OS, MEF and PASMCs	Qi et al., 2008; Wu et al., 2011
C691 S-Nitrosylation	Yes	• Disrupts interaction with GW182 and consequently miRNA mediated repression	HEK-293 and HeLa	Seth et al., 2019
K402 Sumoylation	Only in AGO1	• Destabilizes AGO2 • Increases siRNA activity	HeLa, N2a, MEFs, HT1080	Sahin et al., 2014; Josa-Prado et al., 2015
Ubiquitylation (sites unknown)	Only in AGO2 investigated	• Decreases AGO2 stability • Represses miRNA activity	HEK-293, EC, MEFs, CD4^+^ T, MDA-MB-231	Adams et al., 2009; Rybak et al., 2009; Bronevetsky et al., 2013; Smibert et al., 2013
Poly(ADP-ribose)ylated (sites unknown)	AGO1-4 modified but sites unknown	• Inhibits slicing activity • Reduces RNAi activity	HeLa S3, HEK-293	Leung et al., 2011; Seo et al., 2013
K720, K493, K355 Acetylation	Only in AGO2 investigated	• Recruitment of AGO2 to miR-19b1 precursor	HEK-293T, A549, lung cancer tissue arrays, mouse xenografted tumor model	Zhang et al., 2019
**Phosphorylation**				
S387	Not conserved in AGO3 (others Yes)	• Increases translational repression • Decreases cleavage activity • Reduces sorting into exosomes	HeLa, HEK-293T, HEK-293, DLD1 colon cancer lines, MEFs, U2OS, H1299	Zeng et al., 2008; Rüdel et al., 2011; Horman et al., 2013; Lopez-Orozco et al., 2015; McKenzie et al., 2016; Bridge et al., 2017; Quévillon Huberdeau et al., 2017
Y393	Not conserved in AGO3 (others yes)	•Decreases maturation of AGO2- mediated miRNA under hypoxia • Inhibits loading of miRNA	HEK-293, HEK-293T, HeLa, MDA-MB-231, IMR90	Rüdel et al., 2011; Shen et al., 2013; Yang et al., 2014
Y529	Yes	• Disrupts interaction with mRNA targets and cleavage	HEK-293, HeLa, LPS-activated RAW 264.7, primary macrophages	Rüdel et al., 2011; Mazumder et al., 2013; Lopez-Orozco et al., 2015
S798	Yes	• AGO2 loses its association with P-bodies and stress granules	HeLa	Lopez-Orozco et al., 2015
S253, T303, T307	Yes for S253 and T307, T303 not conserved in AGO4	• Unknown	HEK-293	Rüdel et al., 2011
T555-S561 cluster	Yes	• Impaired localization to P-bodies and silencing	HEK-293T, HeLa	Quévillon Huberdeau et al., 2017
S824-S834 cluster	Yes	• Affects mRNA target association	HEK-293T, HeLa, HCT116	Golden et al., 2017; Quévillon Huberdeau et al., 2017

As previously described, Argonaute proteins, through the formation of a RISC complex, enable miRNAs to downregulate partially complementary target mRNAs, making them relevant in normal physiology and disease. PTMs of AGOs can impact several features of RISC-mediated silencing. For example, a rapid cycle of AGO2 phosphorylation and dephosphorylation of a serine/threonine cluster located on a loop on the surface of the PIWI domain is relevant for miRNA binding to target mRNAs and for miRNA-mediated gene silencing. The dissection of the upstream signaling pathways that impact on AGO2 PTM and, consequently, on its cyclic functional activity would represent a relevant advance in the understanding of AGO2 activity and might possibly provide new ways to modulate the global activity of miRNAs (Golden et al., 2017).

The phosphorylation status of AGOs is also critical for the regulation of the miRNA function in humans (Quévillon Huberdeau et al., 2017). In particular, AGOs are hyperphosphorylated at a C-terminal serine/threonine cluster upon miRNA binding and repression of the mRNA target. The negative charge of phosphates within this region impairs the mRNA/AGO interaction and favors the release of target mRNA. The balance between the phosphorylated and dephosphorylated status of AGO may be relevant also for redirecting AGO to a new target mRNA and for modulation of its degradation (Quévillon Huberdeau et al., 2017).

Furthermore, PTMs of AGOs are involved in miRNA processing. AGO2 phosphorylation has been related to certain cancer phenotypes. In these cases, specific AGO2 phosphorylation leads to reduced interaction between DICER and AGO2 with consequent impairment of miRNA processing (Shen et al., 2013).

In addition, the acetylation of specific lysine residues of AGO2 has been reported. This represents a relevant modification for the recruitment of AGO2 to the miR-19b1 precursor, resulting in the enhancement of oncogenic miR-19b processing. Notably, in lung cancer patients, high levels of both miR-19b and AGO2 acetylation correlate with a poor prognosis (Zhang et al., 2019).

Finally, AGO2 phosphorylation also impacts its localization within the cell. Specific AGO2 phosphorylation has been reported to be essential for its localization into processing bodies (P-bodies), impinging on AGO2-dependent regulation of RNA-silencing activity (Zeng et al., 2008). Horman et al. (2013) subsequently show that AGO2 phosphorylation is critical for the interaction with GW182 protein and AGO2 localization in P-bodies. Furthermore, this modification was also shown to regulate localization of AGO2 into multivesicular endosomes resulting in the suppression of AGO2 secretion and influencing the sorting of specific miRNAs into exosomes (McKenzie et al., 2016).

In summary, PTMs affect several AGO exerted functions. In this paragraph, we have given only a few examples. A broader overview can be found in Table 1.

## The Functions of Argonaute Proteins in Mammalian Early Development

Several studies have examined the roles of AGOs during mouse early development. Earliest works demonstrated that the knockout (KO) of *Ago2* is lethal during early mouse development at postimplantation stages (Liu et al., 2004; Alisch et al., 2007; Morita et al., 2007; Cheloufi et al., 2010). In contrast *Ago1*, *3*, and *4* KO mice are viable (Modzelewski et al., 2012; Van Stry et al., 2012). These studies have shown that *Ago2*-deficient embryos are growth retarded and developmentally delayed. In addition, they display severe phenotypic defects, such as cardiac failure and impaired neuronal tube closure (Liu et al., 2004; Alisch et al., 2007; Cheloufi et al., 2010).

Interestingly, the phenotype of the *Ago2*-deficient mice compared to other RNAi-deficient mice is not identical. In addition to other phenotypic differences, *Dicer-* or *Drosha*-deficient mice, for example, display earlier embryonic lethality compared to *Ago2*-deficient mice, suggesting individual roles for the RNAi effector proteins in regulating embryonic development (Bernstein et al., 2003; Chong et al., 2010).

The function of AGO2 in the embryonic development has already been given ample attention with the help of several mouse models (Liu et al., 2004; Alisch et al., 2007; Morita et al., 2007; Cheloufi et al., 2010). However, detailed analyses of AGO2 in the development of the extraembryonic lineages are still missing. Recently, it has been reported that early mice lethality is often associated with placental defects (Perez-Garcia et al., 2018). Interestingly, previous histological analyses have already indicated that *Ago*2-deficient mice display extraembryonic defects. Supplementing these mice with wild-type extraembryonic tissue is able to rescue the mid-gestation death of *Ago2* KO mice (Liu et al., 2004; Cheloufi et al., 2010). These defects might explain why *Ago*2-deficient mice die at the postimplantation stage; however, this has not been assessed yet.

Ngondo et al. (2018) have recently demonstrated a novel function of AGO2 in the development of the extraembryonic endoderm, *in vitro*. Using mouse embryonic stem cells (mESCs), which are derived from the blastocyst stage, they generated *Ago2* KO mESCs, by CRISPR/Cas9 genome engineering (Wettstein et al., 2016). Upon *in vitro* differentiation, *Ago2* KO mESCs were able to form all three embryonic germ layers; however, they showed impaired differentiation toward the extraembryonic endoderm (Ngondo et al., 2018). This differentiation defect was rescued by the reintroduction of a wild-type AGO2 or a catalytic dead AGO2 in mESCs, but not by an RNA-binding-deficient AGO2 (Ngondo et al., 2018). In line with these results, *Ago2* catalytic dead mice were previously shown to be viable until a few hours after birth and subsequently died of anemia (Cheloufi et al., 2010; Jee et al., 2018). In these cases, the slicing activity of AGO2 is needed to process the pre-miRNA-451 and miRNA-486-3p, two miRNAs required in the development of the erythroblasts (Cheloufi et al., 2010; Papapetrou et al., 2010; Jee et al., 2018).

Notably, the molecular mechanism by which AGO2 regulates the formation of the extraembryonic endoderm still remains elusive. Interestingly, the differentiation defect of the *Ago2* KO mESCs is comparable to what was observed previously for *Gata6* KO mESCs, a key transcription factor required for the formation of the primitive endoderm lineage *in vivo* (Capo-Chichi et al., 2005). Together, these reports indicate a function of AGO2 not only in the development of the embryo proper but also in the extraembryonic lineages.

Mouse embryonic stem cells are a very informative *in vitro* culture system to mimic mouse early development at the blastocyst stage. Nevertheless, a stable *in vitro* system mimicking the earliest stages of development is still missing. Most of the studies focusing on the first cell fate decisions in early mouse development were performed by imaging wild-type or mutant embryos and relied on specific antibodies for the protein of interest, which were not available for a long time for the mouse AGO proteins. Furthermore, single-cell bulk analysis requires a lot of material, which is hard to obtain from early embryos. However, a powerful tool to study the earliest cell fate decision, the two-cell stage-like (2C) ESCs, has been discovered a few years ago (Macfarlan et al., 2012). Two-cell like cells are totipotent and therefore can still differentiate into the extraembryonic as well as embryonic lineages (Baker and Pera, 2018). MESCs have been shown to present a heterogeneous population, where a small subpopulation (<1%) cycles in and out of a two-cell stage (Macfarlan et al., 2012; Morgani et al., 2013; Ishiuchi et al., 2015). The totipotent two-cell stage subpopulation might provide a powerful way to study the impact of AGO2 on the early stages of mouse development, not only for the epiblast but also for the trophoblast lineage, where the impact of AGO2 loss has not been assessed yet. So far, we still do not know whether AGO2 is the only Argonaute protein well expressed in this lineage and whether it impacts trophoblast differentiation.

Since AGO2-deficient mice only die at the postimplantation stage, the question is raised, whether AGO2 is dispensable for preimplantation development or, whether maternally supplied AGO2 regulates these early stages. One important *in vivo* study assessed the requirement of AGO proteins before the blastocyst stage (Lykke-Andersen et al., 2008). Using injection of dsRNAs against maternally supplied Argonautes, they demonstrated that only AGO2 is essential for the development of mouse oocytes to the two-cell stage. Nevertheless, the molecular mechanism by which AGO2 regulates this early transition is still unknown. Strikingly, the loss of another RNAi effector protein in oocytes, DGCR8, displays a very different phenotype compared to the loss of AGO2. *Dgcr8* KO oocytes are able to develop beyond the 2-cell stage to blastocysts. As DGCR8 is only involved in the processing of canonical miRNAs, this suggests that canonical miRNAs might be dispensable for early development (Suh et al., 2010). This is in line with the previous assumptions that miRNA function is lost in oocytes. One reason for the loss of miRNA function in oocytes was proposed to be due to an AGO2-specific oocyte isoform (Freimer et al., 2018). However, a recent report shows that miRNA activity might not be lost in oocytes but that the miRNA/mRNA stoichiometry is impaired in oocytes due to the low abundance of miRNAs (Kataruka et al., 2019). Furthermore, *Ago2* KO oocytes seem very similar to *Dicer* KO oocytes. Both show abnormal spindle and chromosome positioning and fail to undergo the first cleavage to the two-cell stage (Murchison et al., 2007; Tang et al., 2007; Kaneda et al., 2009). Moreover, changes in gene transcripts in *Dicer* KO oocytes are claimed to be provoked by endo-siRNAs (Watanabe et al., 2008), which are the most prominently expressed smRNAs in oocytes and not preprocessed by DGCR8 (Tam et al., 2008; Watanabe et al., 2008; Suh et al., 2010). The loss of DICER and AGO2 in oocytes decreases siRNAs (Watanabe et al., 2008). It is therefore possible that the phenotype observed in *Ago2* KO oocytes is a result of the loss of endo-siRNA-induced target silencing.

Hence, the exact function of AGO2 in early development still needs to be elucidated, as it is undoubtedly the only one leading to a lethal phenotype.

## Conclusion

In this review, we highlight various differences and similarities between the Argonaute proteins to better understand their specialized roles within the cell, especially in regard to AGO2.

With the structural information available nowadays, it is possible to pinpoint the exact residues responsible for the catalytic function or disfunction of the AGOs. This has clarified why AGO2 specifically was thought for a long time to be the only slicer molecule of this family.

Interestingly, however, from available sequencing data, it seems that AGO2 is the only AGO protein well expressed in early mice or human development, at least at the transcriptional level (Boroviak et al., 2018). This, when reflected on protein levels, might also explain the severe defects observed upon the loss of AGO2 in early embryos when compared to AGO1, 3, and 4. Strikingly, the *Ago2* KO phenotypes observed are not just linked to the embryonic development but also cause impairments in extraembryonic development, as studies show placental defects associated with the loss of AGO2 (Liu et al., 2004; Cheloufi et al., 2010). We argue that a deeper exploration in the early development of extraembryonic tissues is warranted in the context of AGO2 loss *in vivo* and *in vitro*.

Lastly, we present an overview of multiple to date known posttranslational modifications of AGO2. These modifications have so far been studied in several cancer models and furthermore have been linked to disease phenotypes. From such studies, we know that PTMs can impact the RISC activity as well as AGO2 stability, either positively or negatively. However, a detailed analysis of such modifications during early development is still missing. We still do not know which modifications are present in early embryos nor whether there is a switch of modification when going through different stages of embryonic or extraembryonic development. Hence, to better understand how AGO2 functions in these early stages of embryonic development, the PTMs of AGO2 must also be taken into consideration.

## Author Contributions

MM and CC wrote the sections related to Argonaute expression and structure in early development. MM drew the figure and established the Table 1. FF contributed to the writing of the section on the PTM of AGO in cancer. All authors read and approved the final version of the manuscript.

## Conflict of Interest

The authors declare that the research was conducted in the absence of any commercial or financial relationships that could be construed as a potential conflict of interest.
